# Proceedings of the 16th Annual UT-KBRIN Bioinformatics Summit 2016: bioinformatics: Burns, TN, USA. April 21-23, 2017

**DOI:** 10.1186/s12859-017-1781-y

**Published:** 2017-10-13

**Authors:** Eric C. Rouchka, Julia H. Chariker, David A. Tieri, Juw Won Park, Shreedharkumar Rajurkar, Vikas Singh, Nishchal K. Verma, Yan Cui, Mark Farman, Bradford Condon, Neil Moore, Jerzy Jaromczyk, Jolanta Jaromczyk, Daniel Harris, Patrick Calie, Eun Kyong Shin, Robert L. Davis, Arash Shaban-Nejad, Joshua M. Mitchell, Robert M. Flight, Qing Jun Wang, Richard M. Higashi, Teresa W-M Fan, Andrew N. Lane, Hunter N. B. Moseley, Liangqun Lu, Bernie J Daigle, Xi Chen, Andrey Smelter, Hunter N. B. Moseley, Jerzy W. Jaromczyk, Mark Farman, Li Chen, Neil Moore, Bailey K. Phan, Nathaniel J. Serpico, Ethan G. Toney, Caroline E. Melton, Jennifer R. Mandel, Bernie J. Daigle, Hao Chen, Kazi I. Zaman, Ramin Homayouni, Patrick J. Trainor, Samantha M. Carlisle, Andrew P. DeFilippis, Shesh N. Rai

**Affiliations:** 1 Department of Computer Engineering and Computer Science, University of Louisville, Duthie Center for Engineering, Louisville, KY 40292 USA; 2 Kentucky Biomedical Research Infrastructure (KBRIN) Bioinformatics Core, 522 East Gray Street, Louisville, KY 40292 USA; 3 Department of Psychological and Brain Sciences, University of Louisville, Louisville, KY 40292 USA; 4 Department of Anatomical Sciences and Neurobiology, University of Louisville, Louisville, KY 40292 USA; 5 Department of Electrical Engineering, IIT Kanpur, India; 6 Department of Microbiology, Immunology and Biochemistry, University of Tennessee Health Science Center, Memphis, TN 38103 USA; 7 Center for Integrative and Translational Genomics, University of Tennessee Health Science Center, Memphis, TN 38103 USA; 8 Department of Plant Pathology, University of Kentucky, Lexington, KY 40546 USA; 9 UK Healthcare Genomics, University of Kentucky, Lexington, KY 40536 USA; 10 Department of Entomology & Plant Pathology, University of Tennessee, Knoxville, TN 37996 USA; 11 Department of Computer Science, University of Kentucky, Lexington, KY 40506 USA; 12 Department of Forestry, University of Kentucky, Lexington, KY 40506 USA; 13 Department of Biological Sciences, Eastern Kentucky University, Richmond, KY 40475 USA; 14 University of Tennessee Health Science Center – Oak-Ridge National Lab (UTHSC-ORNL) Center for Biomedical Informatics, Department of Pediatrics, Memphis, TN 38103 USA; 15 Department of Molecular & Cellular Biochemistry, University of Kentucky, Lexington, KY 40536 USA; 16 Markey Cancer Center, University of Kentucky, Lexington, KY 40536 USA; 17 Center for Environmental and Systems Biochemistry, University of Kentucky, Lexington, KY 40536 USA; 18 Institute for Biomedical Informatics, University of Kentucky, Lexington, KY 40536 USA; 19 Department of Toxicology & Cancer Biology, University of Kentucky, Lexington, KY 40536 USA; 20 Department of Ophthalmology and Visual Sciences, University of Kentucky, Lexington, KY 40536 USA; 21 Department of Biological Sciences, The University of Memphis, Memphis, TN 38152 USA; 22 Department of Computer Science, The University of Memphis, Memphis, TN 38152 USA; 23 Department of Molecular and Cellular Biochemistry, University of Kentucky, Lexington, KY 40536 USA; 24 Department of Statistics, University of Kentucky, Louisville, KY 40536 USA; 25 School of Interdisciplinary and Graduate Studies, University of Louisville, Louisville, KY 40202 USA; 26 Department of Computer Engineering & Computer Science, University of Louisville, Louisville, KY 40202 USA; 27 Markey Cancer Center, University of Kentucky, Lexington, KY 40536 USA; 28 Center for Environmental & Systems Biochemistry, University of Kentucky, Lexington, KY 40536 USA; 29 Institute for Biomedical Informatics, University of Kentucky, Lexington, KY 40536 USA; 30 Department of Computer Science, University of Kentucky, Lexington, KY 40508 USA; 31 Department of Plant Pathology, University of Kentucky, Lexington, KY 40546 USA; 32 Department of Biological Sciences, The University of Memphis, Memphis, TN 38152 USA; 33 Department of Pharmacology, University of Tennessee Health Science Center, Memphis, TN 38103 USA; 34 Department of Computer Science, University of Memphis, Memphis, TN 38152 USA; 35 Department of Biological Sciences, University of Memphis, Memphis, TN 38152 USA; 36 Bioinformatics Program, University of Memphis, Memphis, TN 38152 USA; 37 Department of Medicine, Division of Cardiovascular Medicine, University of Louisville, Louisville, KY 40202 USA; 38 Diabetes and Obesity Center, University of Louisville, Louisville, KY 40202 USA; 39 Department of Pharmacology and Toxicology, University of Louisville, Louisville, KY 40202 USA; 40 Biostatistics Shared Facility, James Graham Brown Cancer Center, University of Louisville, Louisville, KY 40202 USA; 41 Department of Bioinformatics and Biostatistics, University of Louisville, Louisville, KY 40202 USA

## I1 Proceedings of the Sixteenth Annual UT- KBRIN Bioinformatics Summit 2017

### Eric C Rouchka^1,2^, Julia H Chariker^2,3^, David A Tieri^2,4^, Juw Won Park^1,2^

#### ^1^Department of Computer Engineering and Computer Science, University of Louisville, Duthie Center for Engineering, Louisville, KY 40292, USA; ^2^Kentucky Biomedical Research Infrastructure (KBRIN) Bioinformatics Core, 522 East Gray Street, Louisville, KY 40292, USA; ^3^Department of Psychological and Brain Sciences, University of Louisville, Louisville, KY 40292, USA; ^4^Department of Anatomical Sciences and Neurobiology, University of Louisville, Louisville, KY 40292, USA

##### **Correspondence:** Eric C Rouchka (eric.rouchka@louisville.edu)

**Background**

The University of Tennessee (UT) and the Kentucky Biomedical Research Infrastructure Network (KBRIN) have collaborated over the past sixteen years to share research and educational expertise in bioinformatics. One result is an annual regional summit for researchers, educators, and students. The Sixteenth Annual UT- KBRIN Bioinformatics Summit was held at Montgomery Bell State Park in Burns, Tennessee from April 21-23, 2017. A total of 243 participants pre-registered, with 113 from Kentucky, 111 from Tennessee, and the remainder from various states and international locales. Among the registrants were 93 students, 90 faculty, 39 staff, and 21 postdocs. The conference program consisted of two workshops on R, a free software environment for statistical computing and graphics, and two days of plenary presentations and short talks. In addition, a poster session with 40 posters was held on Saturday evening.

**Friday Workshops**

The University of Kentucky R Team opened the Summit with a two-part workshop. The first part of the workshop, “Introduction to Data Analysis with R”, led by Katherine Thompson and Arnold Stromberg, focused on some basics of using the R language. Among the topics covered were opening data in R, visualizing data, and some basic data analyses in R. Part 2 of the workshop, “How to Use and Create Interactive Shiny Applications”, led by Joshua Lambert, covered how to create Shiny applications. These applications allow for the construction of web-based interfaces to allow users to run R software without programming or installing R.

**Session I: Biomedical Informatics**

The Summit keynote speaker, Peter Laussen (University of Toronto), opened the first scientific session, Biomedical Informatics, on Saturday morning with a presentation on “Reducing Modifiable Risk in Critical Care: The Promise of Harnessing Physiologic Data Streams.” This presentation focused on integrating technology and data scientists with clinicians to provide both a safe and efficient patient journey through their care. Dr. Laussen focused on some specific examples where real time integration of data science is likely to have a positive influence on both clinical care and cost reduction in the context of cardiac critical care [1, 2]. He explored many of the challenges with this data, and discussed techniques for visualizing the data with methods similar to those used by NASCAR to monitor drivers during a race.

Oguz Akbilgic (University of Tennessee Health Science Center - UTHSC) followed with a talk titled “Probabilistic Symbolic Pattern Recognition (PSPR) in Clinical Decision Making.” This presentation focused on the use of the PSPR method for identifying predictors of pathophysiology from a number of clinical features. He gave a specific example of their use in detecting paroxysmal atrial fibrillation using clustering of ECGs.

Arash Shaban-Nejad (UTHSC) continued the session with the presentation “Urban Health Intelligence for Public Health Planning and Policy Development.” This talk discussed the correlations between socioeconomic status and population health. Dr. Shaban-Nejad discussed PopHR [3], a knowledge-based platform for integrating, analyzing, and visualizing population health data.

Rishi Kamalsweran (University of Tennessee Health Science Center) closed the Biomedical Informatics Session with the presentation “Dynamic Visual Analytics and Event Stream Processing.” In this talk, Dr. Kamalsweran discussed the prospects of bringing analytics to the bedside in order to predict the onset of disease. A number of approaches that have been developed for bringing earlier, personalized care to patients [4-6] were discussed in addition to methods for visualizing dynamic streaming data [7].

**Session II: Systems Biology**

Session II: Systems Biology began with a presentation by Qui Liu (Vanderbilt University) on “Translating Multi-Omics Data into Colorectal Cancer Biology.” In this presentation, Dr. Liu focused on the two aims of integration of multi-dimensional data, including understanding the relationships between the different types of data and understanding both the latent and observable phenotypes. She discussed integrative techniques they have used for transcriptomics, proteomics, and miRNA [8] as well as other methods for integrating multi-omics data with clinical applications, such as the determination of the cause of resistance to chemotherapeutics in colon cancer [9, 10].

Bruce Ramshaw (University of Tennessee, Knoxville) followed with the presentation “Complex Systems Science Applied to Health Care.” Dr. Ramshaw discussed how many of the issues with health care today are due to a reductionist view, which leads to increasing fragmentation and administration. He suggested that rather than view clinical health care through a reductionist view, a complex systems science view is needed in order to change the assumptions and resulting tools for clinicians and clinical researchers. He showed how implementation of such a collaborative team led to substantial savings in a health care system due to decreased length in post-operative stay and reduction in material costs [11].

The third speaker in the Systems Biology section, Rachel McCord (University of Tennessee-Knoxville), presented “The 3D Genome: Folding, Misfolding, and Unfolding.” In this talk, Dr. McCord discussed how DNA folding leads to biological function when the genome folds itself into a 3-dimensional shape, leading to a number of different interactions, or 3D compartments, between chromosomes. In cancer and other diseases, translocataions interrupt these interactions. She also introduced the methods they have employed for measuring genome folding, including Hi-C [12, 13]. These techniques were used to study the loss of 3D genome compartments in progeria patients [14].

David Ussery (University of Arkansas for Medical Sciences) continued with the presentation “What can 100,000 Bacterial Genomes Teach Us about Evolution?” in which he discussed the genetic diversity in the bacterial genome, and showed that no single protein is conserved among all living organisms, but that functional domains are conserved [15]. This analysis has been made possible through 20 years of bacterial genome sequencing [16] as well as increased availability of high throughput sequencers such as the Minion nanopore sequencers.

Robert Flight (University of Kentucky) finished off the Systems Biology session with his presentation “Meta- and Multi-Omic Analyses Using Annotations.” In this presentation, Dr. Flight discussed the use of annotation enrichment, which can be applied to various –omics data sets, for analysis of a particular phenotype. He discussed categoryCompare [17] an approach he developed for such analysis, and its extended version to show its utility for analyzing the effects of three different gene knockouts on Juvenile Batten Disease.

**Session III: Metabolomics**

Richard Higashi (University of Kentucky) kicked off the Metabolomics session on Sunday morning. During his presentation, Dr. Higashi discussed methods developed for measuring the flux of labeled metabolites through a system, and the corresponding issues with modeling such flux as well as visualizing the resulting data sets [18-23] with a specific example of cancer.

Christine Fillmore Brainson (University of Kentucky) closed the Metabolomics session with a presentation “Integrating Epigenetics, Transcriptomics and Metabolomics Datasets from a Lung Cancer Model.” This presentation, which takes a systems-approach to modeling disease, looked at how different tri-methylation events are affected by carcinomas and how they correlate with transcription. In addition, she discussed how metabolism affects stability.

**Session IV: Single Cell Omics and Other NGS**

The final scientific session of the summit focused on the use of high-throughput sequencing datasets to analyze data in such a way that could not previously be studied. Corey Watson (University of Louisville) began this session with a talk “Genomics of the Functional Antibody Response in Human.” During this presentation, Dr. Watson discussed the high degree of variability determined within the IgH region, both in terms of longer variants and SNPs, and how high throughput sequencing can be used to resolve some of these [24-28]. He discussed how these regions also have high variation in copy number, and conveyed how his lab is beginning to use long sequencing reads to address issues with reassembling this highly variable region.

Eric Rouchka (University of Louisville) followed with the presentation “Identification of Cleavage Site Intervals for Alternative 3’ UTR dynamics.” During this presentation, Dr. Rouchka discussed development of an algorithm for detecting 3’ UTR lengthening and shortening events [29], which was motivated by some previous findings for localization based on alternative 3’ UTR usage [30] and detection of a number of alternative 3’ UTR events within nervous system processes [31, 32].

Arthur Hunt (University of Kentucky) followed with the presentation “The Intersection of Alternative Polyadenylation and RNA Quality Control.” During this presentation, Dr. Hunt described the prevalence of polyadenylation within plant genomes [33-35]. He also introduced methods his lab has developed for inexpensive library construction for high-throughput sequencing [36, 37].

Juw Won Park (University of Louisville) ended the session with his talk on alternative splicing and circular RNAs. He showed that an organism’s protein diversity is not determined solely by the number of genes it possesses, but also by its ability to utilize alternative splicing of its genes. It was also shown that circular RNAs, which participate in biological function such as gene regulation via modulating microRNAs activity [38], can exhibit alternative splicing [39]. He discussed the software that he developed that can detect differential alternative splicing events from RNA-Seq data [40-42]. He also introduced an approach that can estimate the abundance of circular RNAs with respect to linear forms from RNA-Seq data.

**Poster Session**

A poster session and reception was held on Saturday evening with a total of 41 posters presented across 14 categories. The largest represented categories included transcriptomics, bioinformatics algorithms, phylogenetics, protein structure and proteomics, and systems biology and networks. Nineteen of the poster abstracts along with one speaker abstract are highlighted within this supplement. Prior to the poster session, 34 of the posters were presented during a one-minute blitz session used to introduce the posters and their topics.

**Acknowledgements**

We would like to thank the Conference Program Committee members Hao Chen (University of Tennessee Health Science Center), Nigel Cooper (University of Louisville), Dan Goldowitz (University of British Columbia), Mike Langston (University of Tennessee-Knoxville), Terry Mark-Major (University of Tennessee Health Science Center), Hunter Moseley (University of Kentucky), Juw Won Park (University of Louisville), Claire Rinehart (Western Kentucky University), Arnold Stromberg (University of Kentucky), and Rob Williams (University of Tennessee Health Science Center) for organizing an outstanding scientific program. In addition, we wish to thank Susan Boucher, Tamara Brock, Terry Mark-Major, Michelle Padgett, and Whitney Rogers for their efforts in handling conference organization details. Funding for the UT- KBRIN Summit is provided in part by the University of Memphis Office of the Provost, Memphis Research Consortium, Kentucky Biomedical Research Infrastructure Network (KBRIN), University of Tennessee Center for Integrative and Translational Genomics, University of Tennessee Molecular Resource Center, and NIH grant P20GM103436.

**References**

1. Checchia PA, Laussen PC, Macrae D, Bohn D, Chang AC, Wessel DL: **Pediatric Cardiac Intensive Care: A Transition to Maturity**. *Pediatr Crit Care Med* 2016, **17**(8 Suppl 1):S110-111.

2. Smith AH, Laussen PC: **Cardiac critical care: what really makes a difference**. *Curr Opin Pediatr* 2013, **25**(5):567-573.

3. Shaban-Nejad A, Lavigne M, Okhmatovskaia A, Buckeridge DL: **PopHR: a knowledge-based platform to support integration, analysis, and visualization of population health data**. *Ann N Y Acad Sci* 2017, **1387**(1):44-53.

4. Kamaleswaran R, McGregor C: **Integrating complex business processes for knowledge-driven clinical decision support systems**. *Conf Proc IEEE Eng Med Biol Soc* 2012, **2012**:1306-1309.

5. Kamaleswaran R, McGregor C, Eklund J: **A method for clinical and physiological event stream processing**. *Conf Proc IEEE Eng Med Biol Soc* 2010, **2010**:1170-1173.

6. Kamaleswaran R, McGregor C, Percival J: **Service oriented architecture for the integration of clinical and physiological data for real-time event stream processing**. *Conf Proc IEEE Eng Med Biol Soc* 2009, **2009**:1667-1670.

7. Kamaleswaran R, McGregor C: **A Review of Visual Representations of Physiologic Data**. *JMIR Med Inform* 2016, **4**(4):e31.

8. Liu Q, Halvey PJ, Shyr Y, Slebos RJ, Liebler DC, Zhang B: **Integrative omics analysis reveals the importance and scope of translational repression in microRNA-mediated regulation**. *Mol Cell Proteomics* 2013, **12**(7):1900-1911.

9. Li C, Singh B, Graves-Deal R, Ma H, Starchenko A, Fry WH, Lu Y, Wang Y, Bogatcheva G, Khan MP *et al*: **Three-dimensional culture system identifies a new mode of cetuximab resistance and disease-relevant genes in colorectal cancer**. *Proc Natl Acad Sci U S A* 2017, **114**(14):E2852-E2861.

10. Shyr D, Liu Q: **Next generation sequencing in cancer research and clinical application**. *Biol Proced Online* 2013, **15**(1):4.

11. Stephan B, Ramshaw B, Forman B: **Value-based Clinical Quality Improvement (CQI) for Patients Undergoing Abdominal Wall Reconstruction**. *Surg Technol Int* 2015, **26**:135-142.

12. Belton JM, McCord RP, Gibcus JH, Naumova N, Zhan Y, Dekker J: **Hi-C: a comprehensive technique to capture the conformation of genomes**. *Methods* 2012, **58**(3):268-276.

13. Imakaev M, Fudenberg G, McCord RP, Naumova N, Goloborodko A, Lajoie BR, Dekker J, Mirny LA: **Iterative correction of Hi-C data reveals hallmarks of chromosome organization**. *Nat Methods* 2012, **9**(10):999-1003.

14. McCord RP, Nazario-Toole A, Zhang H, Chines PS, Zhan Y, Erdos MR, Collins FS, Dekker J, Cao K: **Correlated alterations in genome organization, histone methylation, and DNA-lamin A/C interactions in Hutchinson-Gilford progeria syndrome**. *Genome Res* 2013, **23**(2):260-269.

15. Ussery DW, Wassenaar TM, Borini S: **Computing for comparative microbial genomics: bioinformatics for microbiologists**, vol. 8: Springer Science & Business Media; 2009.

16. Land M, Hauser L, Jun SR, Nookaew I, Leuze MR, Ahn TH, Karpinets T, Lund O, Kora G, Wassenaar T *et al*: **Insights from 20 years of bacterial genome sequencing**. *Funct Integr Genomics* 2015, **15**(2):141-161.

17. Flight RM, Harrison BJ, Mohammad F, Bunge MB, Moon LD, Petruska JC, Rouchka EC: **categoryCompare, an analytical tool based on feature annotations**. *Front Genet* 2014, **5**:98.

18. Fan TW, Lorkiewicz PK, Sellers K, Moseley HN, Higashi RM, Lane AN: **Stable isotope-resolved metabolomics and applications for drug development**. *Pharmacol Ther* 2012, **133**(3):366-391.

19. Fan TW, Warmoes MO, Sun Q, Song H, Turchan-Cholewo J, Martin JT, Mahan A, Higashi RM, Lane AN: **Distinctly perturbed metabolic networks underlie differential tumor tissue damages induced by immune modulator beta-glucan in a two-case ex vivo non-small-cell lung cancer study**. *Cold Spring Harb Mol Case Stud* 2016, **2**(4):a000893.

20. Lane AN, Higashi RM, Fan TW: **Preclinical models for interrogating drug action in human cancers using Stable Isotope Resolved Metabolomics (SIRM)**. *Metabolomics* 2016, **12**(7).

21. Lane AN, Tan J, Wang Y, Yan J, Higashi RM, Fan TW: **Probing the metabolic phenotype of breast cancer cells by multiple tracer stable isotope resolved metabolomics**. *Metab Eng* 2017.

22. Moseley HN, Lane AN, Belshoff AC, Higashi RM, Fan TW: **A novel deconvolution method for modeling UDP-N-acetyl-D-glucosamine biosynthetic pathways based on (13)C mass isotopologue profiles under non-steady-state conditions**. *BMC Biol* 2011, **9**:37.

23. Sud M, Fahy E, Cotter D, Azam K, Vadivelu I, Burant C, Edison A, Fiehn O, Higashi R, Nair KS *et al*: **Metabolomics Workbench: An international repository for metabolomics data and metadata, metabolite standards, protocols, tutorials and training, and analysis tools**. *Nucleic Acids Res* 2016, **44**(D1):D463-470.

24. Avnir Y, Watson CT, Glanville J, Peterson EC, Tallarico AS, Bennett AS, Qin K, Fu Y, Huang CY, Beigel JH *et al*: **IGHV1-69 polymorphism modulates anti-influenza antibody repertoires, correlates with IGHV utilization shifts and varies by ethnicity**. *Sci Rep* 2016, **6**:20842.

25. Watson CT, Breden F: **The immunoglobulin heavy chain locus: genetic variation, missing data, and implications for human disease**. *Genes Immun* 2012, **13**(5):363-373.

26. Watson CT, Matsen FAt, Jackson KJL, Bashir A, Smith ML, Glanville J, Breden F, Kleinstein SH, Collins AM, Busse CE: **Comment on "A Database of Human Immune Receptor Alleles Recovered from Population Sequencing Data"**. *J Immunol* 2017, **198**(9):3371-3373.

27. Watson CT, Steinberg KM, Graves TA, Warren RL, Malig M, Schein J, Wilson RK, Holt RA, Eichler EE, Breden F: **Sequencing of the human IG light chain loci from a hydatidiform mole BAC library reveals locus-specific signatures of genetic diversity**. *Genes Immun* 2015, **16**(1):24-34.

28. Watson CT, Steinberg KM, Huddleston J, Warren RL, Malig M, Schein J, Willsey AJ, Joy JB, Scott JK, Graves TA *et al*: **Complete haplotype sequence of the human immunoglobulin heavy-chain variable, diversity, and joining genes and characterization of allelic and copy-number variation**. *Am J Hum Genet* 2013, **92**(4):530-546.

29. Harrison BJ, Flight RM, Eteleeb AM, Rouchka EC, Petruska JC: **UTR extension and alternate polyadenylation in neuroplasticity: an emerging paradigm?** *BMC Bioinformatics* 2014, **15**(10):P11.

30. Harrison BJ, Flight RM, Gomes C, Venkat G, Ellis SR, Sankar U, Twiss JL, Rouchka EC, Petruska JC: **IB4-binding sensory neurons in the adult rat express a novel 3' UTR-extended isoform of CaMK4 that is associated with its localization to axons**. *J Comp Neurol* 2014, **522**(2):308-336.

31. Harrison BJ, Venkat G, Hutson T, Rau KK, Bunge MB, Mendell LM, Gage FH, Johnson RD, Hill C, Rouchka EC *et al*: **Transcriptional changes in sensory ganglia associated with primary afferent axon collateral sprouting in spared dermatome model**. *Genom Data* 2015, **6**:249-252.

32. Harrison BJ, Venkat G, Lamb JL, Hutson TH, Drury C, Rau KK, Bunge MB, Mendell LM, Gage FH, Johnson RD *et al*: **The Adaptor Protein CD2AP Is a Coordinator of Neurotrophin Signaling-Mediated Axon Arbor Plasticity**. *J Neurosci* 2016, **36**(15):4259-4275.

33. Bell SA, Shen C, Brown A, Hunt AG: **Experimental Genome-Wide Determination of RNA Polyadenylation in Chlamydomonas reinhardtii**. *PLoS One* 2016, **11**(1):e0146107.

34. Chakrabarti M, Hunt AG: **CPSF30 at the Interface of Alternative Polyadenylation and Cellular Signaling in Plants**. *Biomolecules* 2015, **5**(2):1151-1168.

35. Wu X, Liu M, Downie B, Liang C, Ji G, Li QQ, Hunt AG: **Genome-wide landscape of polyadenylation in Arabidopsis provides evidence for extensive alternative polyadenylation**. *Proc Natl Acad Sci U S A* 2011, **108**(30):12533-12538.

36. Hunt AG: **A rapid, simple, and inexpensive method for the preparation of strand-specific RNA-Seq libraries**. *Methods Mol Biol* 2015, **1255**:195-207.

37. Ma L, Hunt AG: **A 3' RACE protocol to confirm polyadenylation sites**. *Methods Mol Biol* 2015, **1255**:135-144.

38. Xu H, Guo S, Li W, Yu P: **The circular RNA Cdr1as, via miR-7 and its targets, regulates insulin transcription and secretion in islet cells**. *Sci Rep* 2015, **5**:12453.

39. Zhang XO, Wang HB, Zhang Y, Lu X, Chen LL, Yang L: **Complementary sequence-mediated exon circularization**. *Cell* 2014, **159**(1):134-147.

40. Park JW, Tokheim C, Shen S, Xing Y: **Identifying differential alternative splicing events from RNA sequencing data using RNASeq-MATS**. *Methods Mol Biol* 2013, **1038**:171-179.

41. Shen S, Park JW, Huang J, Dittmar KA, Lu ZX, Zhou Q, Carstens RP, Xing Y: **MATS: a Bayesian framework for flexible detection of differential alternative splicing from RNA-Seq data**. *Nucleic Acids Res* 2012, **40**(8):e61.

42. Shen S, Park JW, Lu ZX, Lin L, Henry MD, Wu YN, Zhou Q, Xing Y: **rMATS: robust and flexible detection of differential alternative splicing from replicate RNA-Seq data**. *Proc Natl Acad Sci U S A* 2014, **111**(51):E5593-5601.

## P1 Deep neural network with fuzzy inference system for transcriptome-based cancer classification

### Shreedharkumar Rajurkar^1^, Vikas Singh^1^, Nishchal K. Verma^1^, Yan Cui^2,3^

#### ^1^Department of Electrical Engineering, IIT Kanpur, India; ^2^Department of Microbiology, Immunology and Biochemistry, University of Tennessee Health Science Center, Memphis, TN 38103, USA; ^3^Center for Integrative and Translational Genomics, University of Tennessee Health Science Center, Memphis, TN 38103, USA

##### **Correspondence:** Yan Cui (ycui2@uthsc.edu)

**Background**

In recent years, knowledge extraction from the biomedical data has become major challenge [1]. Machine learning has presented advanced tools for representation learning in biomedical field. But the performance of conventional machine learning algorithms is feature dependent. These features are designed by a human expert in those domains, and identifying which features are more appropriate for the given task remains a difficult problem. Deep learning is an advancement in machine leaning to deal with such a problem.

**Materials and methods**

We have used a deep neural network using a Takagi-Sugeno fuzzy inference system to learn data representation in the form of fuzzy structures [2]. A generic architecture built from connecting layers of Takagi-Sugeno fuzzy inference system as nodes is elaborated and various parameters involved in it are discussed. This architecture has an input layer, multiple hidden layers and an output layer. But the last two layers of the network have a Takagi-Sugeno fuzzy inference system as its fundamental building unit [3]. Training is carried out using gradient descent to achieve the identification of all parameters in the architecture according to training data. The proposed architecture is implemented in two class Prostate Cancer data [4] containing 102 samples and 10509 genes. Individual training error ranking is used for selecting best features. These features are then passed to the network to learn intricate fuzzy representation in the form of multiple distinct fuzzy rule bases which are intelligible to a human. The identified fuzzy rule bases consist of linguistic information of IF-THEN rules which may turn out to be helpful in diagnosis of disease at the time of examination of patient.

**Results and conclusions**

The result of the proposed network is compared on the basis of AUC (Area under ROC curve) performance with respect to deep learning model using neural network with softmax fine tuning. The use of Takagi-Sugeno fuzzy inference system may improve the performance of deep neural network on transcriptome-based cancer classification.

**References**

1. Min S, Lee B, Yoon S: **Deep learning in bioinformatics.** *Briefings in Bioinformatics*. 2016; bbw068. doi: 10.1093/bib/bbw068

2. Zadeh L A: **Fuzzy sets**. *Information and Control*. 1965; 8(3):338-353. doi: 10.1016/S0019-9958(65)90241-X

3. Yen J, Wang L and Gillespie CW: **Improving the interpretability of TSK fuzzy models by combining global learning and local learning**. *IEEE Transactions on Fuzzy Systems.*1998; 6(4):530-537. doi: 10.1109/91.728447

4. Singh D, Febbo PG, Ross K, Jackson DG, Manola J, Ladd C, Tamayo P, Renshaw AA, D'Amico AV, Richie JP, Lander ES: **Gene expression correlates of clinical prostate cancer behavior.** *Cancer Cell.* 2002; 1(2):203-209. doi: 10.1016/S1535-6108(02)00030-2

## P2 The KBRIN summer workshop: intensive training in Next Gen Sequencing and bioinformatics for novices

### Mark Farman^1,2^, Bradford Condon^1, 3^, Neil Moore^4^, Jerzy Jaromczyk^4^, Jolanta Jaromczyk^1,5^, Daniel Harris^4^, Patrick Calie^6^

#### ^1^Department of Plant Pathology, University of Kentucky, Lexington, KY 40546, USA; ^2^UK Healthcare Genomics, University of Kentucky, Lexington, KY 40536, USA; ^3^Department of Entomology & Plant Pathology, University of Tennessee, Knoxville, TN 37996, USA; ^4^Department of Computer Science, University of Kentucky, Lexington, KY 40506, USA; ^5^Department of Forestry, University of Kentucky, Lexington, KY 40506, USA; ^6^Department of Biological Sciences, Eastern Kentucky University, Richmond, KY 40475, USA

##### **Correspondence:** Patrick Calie (pat.calie@eku.edu)

This abstract is not included here as it has already been published.

## P3 A population health analytics platform for exploring neighborhood effects on distribution of pediatric asthma in Memphis, Tennessee

### Eun Kyong Shin^1^, Robert L Davis^1^, Arash Shaban-Nejad^1^

#### ^1^University of Tennessee Health Science Center – Oak-Ridge National Lab (UTHSC-ORNL) Center for Biomedical Informatics, Department of Pediatrics, Memphis, TN 38103, USA

##### **Correspondence:** Eun Kyong Shin (eshin3@uthsc.edu); Arash Shaban-Nejad (ashabann@uthsc.edu)

**Background**

Socio-economic risk factors -race, urban residence and poverty- significantly contribute to pediatric asthma prevalence [1-3]. However, direct assessments on built-environment and neighborhood effects have not been thoroughly examined due to the scarcity and heterogeneity of available data. According to the theory of Social determinants of health [4], in order to systematically analyze the prevalence of asthma in children and understand its underlying etiology, direct examination of residential factors is crucial. Using knowledge-based platforms enables integration of multiple data sources into a smart and consistent population health surveillance system [5].

**Methods and Results**

Using a knowledge-based population health analytics platform we compare localized pediatric asthma prevalence in 32 zip-code areas in Memphis, TN, combining 6,538 encounter data from Le Bonheur children’s hospital in Memphis and Shelby County Health Department, US census data, and neighborhood quality survey data provided from our partners. Expanding the existing socio-economic models, we explore the neighborhood effects on localized asthma prevalence, which is measured through a number of pediatric asthma encounters observed in each zip-code area. We find that asthma encounters are disproportionately distributed. We use the population size of each zip-code area as control variable. Poverty is known to have a positive association with asthma in the U.S. [4]. Thus, we include the poverty ratio of each zip-code area into socio-economic model. Furthermore, the encounter data shows that 86.2% of encounters are associated with African-American and 8.5% cases are of White. Correspondingly, we add the African-American population ratio into the socio-economic model to control the unique composition of the urban area and our sample. For the neighborhood model, we introduce blight and broken window variables whether living condition of the neighborhood have significant influence to the degree of pediatric asthma prevalence. To predict the prevalence, we run multivariable regression models (Table 1). The base-line model agrees with previous studies showing that the poverty level is positively associated with the asthma prevalence. However, when the racial factor is introduced, it loses its statistical significance. Furthermore, in the neighborhood models, blight phenomenon and broken window variables are positively and significantly associated with the prevalence even after controlling all socio-economic variables. We found that the asthma prevalence is more sensitive to environments. Explanatory power of neighborhood models also increases to 77% approximately.

**Table 1 (abstract P3).** Multivariate regression models on asthma prevalence

	Socio-Economic I		Socio-Economic II		Neighborhood I		Neighborhood II	
Population	.008	***	-.00009		-.00042		.00002	
Poverty level	702.72	***	325.50		114.18		137.41	
African American			.00927	**	.00746	**	.00782	**
Blight					.18930	**		
Broken Windows							.74535	**
Constant	-230.305	**	-55.856		-23.398		-34.671	
Adj R2	0.5890		0.7002		0.7671		0.7652	

NOTE: *p < .05; **p < .01; ***p < 0.001

**Conclusions**

The integration of multiple data sources allows us to unpack the systematic prevalence patterns and broaden our comprehension of asthma epidemic in urban area. Pediatric Asthma is disproportionately prevalent in poor and bad quality neighborhood. Using the socio-environmental indicators public health organizations can implement intelligent surveillance systems for neighborhood-level monitoring of major upstream determinants of health. It is worth mentioning that our sample is an exaggerated composition considering that 53.5% of African American and 42.0% of White at the city-level racial composition (2015 Census) and, therefore, much caution is needed when making inferences about broader contexts. We are in the process of acquiring additional data sets to resolve the current limitation of the study.

**References**

1. Beasley R, Crane J, Lai CK, Pearce N: **Prevalence and etiology of asthma**. *Journal of Allergy and Clinical Immunology*. 2000; 105(2):S466-S472. doi: 10.1016/S0091-6749(00)90044-7

2. Andrew Aligne C, Auinger P, Byrd RS, Weitzman M: **Risk factors for pediatric asthma: contributions of poverty, race, and urban residence**. *American journal of respiratory and critical care medicine*. 2000; 162(3):873-877. doi: 10.1164/ajrccm.162.3.9908085

3. Kattan M, Mitchell H, Eggleston P, Gergen P, Crain E, Redline S, Weiss K: **Characteristics of inner-city children with asthma**. *Pediatr Pulmonol*. 1997; 24:253-262.

4. Mikkonen J, Raphael D: **Social determinants of health: The Canadian facts**. 2010. York University, School of Health Policy and Management.

5. Shaban-Nejad, A., Lavigne, M., Okhmatovskaia, A., and Buckeridge, D.L. **PopHR: a knowledge-based platform to support integration, analysis, and visualization of population health data.** Ann N Y Acad Sci. 2017 1387(1), pp. 44-53.

## P4 Detection and handling of spectral artifacts in Fourier transform mass spectra of metabolomics experiments

### Joshua M Mitchell^1,2,3^, Robert M Flight^2,3^, Qing Jun Wang^2,6^, Richard M Higashi^2,3,5^, Teresa W-M Fan^2,3,5^, Andrew N Lane^2,3,5^, Hunter NB Moseley^1,2,3,4^

#### ^1^Department of Molecular & Cellular Biochemistry, University of Kentucky, Lexington, KY 40536, USA; ^2^Markey Cancer Center, University of Kentucky, Lexington, KY 40536, USA; ^3^Center for Environmental and Systems Biochemistry, University of Kentucky, Lexington, KY 40536, USA; ^4^Institute for Biomedical Informatics, University of Kentucky, Lexington, KY 40536, USA; ^5^Department of Toxicology & Cancer Biology, University of Kentucky, Lexington, KY 40536, USA; ^6^Department of Ophthalmology and Visual Sciences, University of Kentucky, Lexington, KY 40536, USA

##### **Correspondence:** Hunter NB Moseley (hunter.moseley@uky.edu)

**Background**

Direct infusion Fourier-transform mass spectrometry (FTMS) allows for high-throughput detection of thousands of metabolites. Typically, the majority of the observed spectral features does not correspond to known metabolites and thus cannot be placed into existing metabolic networks. Without accurate metabolite assignment, discerning their roles in biological systems is not possible. MS Assignment remains difficult due to the low abundance of some detected metabolites, the volume of data produced by FTMS, the small m/z differences between isotopologues, and the lack of sufficient chemical structural information. Additional phenomena producing large numbers of spectral artifacts further complicate FTMS assignment. False assignments including those made on artifact peaks can create large interpretative errors.

**Materials and Methods**

Through manual inspection of FTMS spectra, we identified FTMS-unique artefacts that result in regions of abnormally high peak density (HPD) that we collectively refer to as HPD artefacts. We have implemented an algorithm in Python3 to identify regions of spectra with the HPD property and likely contain a large number of artefactual peaks. First, our algorithm divides a spectrum into a number of overlapping chunks approximately 1 m/z in width and for each window, the peak density is calculated (number of peaks/window width in m/z). Second, the peak density of each chunk is then compared against the peak density of neighboring sets of chunks and a modified chi-squared statistic calculated for each comparison. High statistic values correspond to regions of spectra with the HPD property. This approach robustly identifies HPD artefacts and is tolerant to changes in signal-to-noise, peak densities, etc. that can vary between different FTMS instruments and experimental designs. Once identified, these artefacts can be excluded from subsequent analyses. However, in the case that HPD artefact location correlates with sample class or other experimental variable, more complex methods of artefact removal must be employed to avoid confounds and additional interpretative errors.

**Results and conclusions**

Using our HPD detector, we have identified three types of HPD artefacts:: i) fuzzy sites representing small regions of m/z space with a ‘fuzzy’ appearance due to the extremely high number of peaks present; ii) ringing due to a very intense peak producing side bands of decreasing intensity that are symmetrically distributed around the main peak; and iii) partial ringing where only a subset of the side bands are observed for an intense peak. Fuzzy sites and partial ringing appear to be novel artifacts previously unreported in the literature and we hypothesize that all three artifact types derive from Fourier transformation-based issues. We have developed a set of tools to detect these artifacts and are developing new methods to mitigate or eliminate their effects on FTMS spectra and downstream analyses.

## P5 Large-scale microarray data based feature selection for improved molecular classification

### Liangqun Lu^1^, Bernie J Daigle, Jr.^1,2^

#### ^1^Department of Biological Sciences, The University of Memphis, Memphis, TN 38152, USA; ^2^Department of Computer Science, The University of Memphis, Memphis, TN 38152, USA

##### **Correspondence:** Bernie J Daigle, Jr (bjdaigle@memphis.edu)

**Background**

Supervised feature selection for high-dimensional biological data is a critical component in the development of accurate diagnostic/prognostic molecular classifiers for complex diseases. Wrapper methods and other embedded techniques closely linked to learning algorithms have been widely applied to this task, while feature selection methods incorporating prior biological knowledge are less commonly used. However, these *knowledge-driven* methods have the potential to simultaneously improve classification performance as well as model interpretability.

**Materials and methods**

We adopted a Bayesian strategy for knowledge-driven feature selection to improve gene expression-based classification. By collecting and analyzing microarray gene expression profiles across hundreds of thousands of samples from the Gene Expression Omnibus (GEO), we have estimated prior probabilities of differential expression for each gene in the human genome. Using these probabilities, we have created a novel feature selection scheme based on the empirical Bayesian *limma* framework. Use of this knowledge-driven approach leads to the selection of qualitatively different features compared to those selected by knowledge-agnostic approaches.

**Results**

We have applied our feature selection approach to two publicly available gene expression datasets studying leukemia and asthma. Using both our knowledge-driven feature selection approach as well as a knowledge-agnostic method, we applied supervised support vector machine and logistic regression classifiers. We evaluated classification performance by measuring the area under the receiver operating characteristic curve (AUC). In the asthma dataset, our preliminary results suggest an improvement in AUC resulting from knowledge-driven feature selection. Current work involves applying our method to additional high-dimensional datasets, including recently collected data interrogating posttraumatic stress disorder (PTSD).

**Acknowledgements**

Research was sponsored in part by the Army Research Laboratory and was accomplished under Grant Number W911NF-17-1-0069.

## P6 Protein NMR reference correction: a statistical solution to an analytical problem

### Xi Chen^1,2^, Andrey Smelter^3,4^, Hunter NB Moseley^1,2,5,6,7^

#### ^1^Department of Molecular and Cellular Biochemistry, University of Kentucky, Lexington, KY 40536, USA; ^2^Department of Statistics, University of Kentucky, Louisville, KY 40536, USA; ^3^School of Interdisciplinary and Graduate Studies, University of Louisville, Louisville, KY 40202, USA; ^4^Department of Computer Engineering & Computer Science, University of Louisville, Louisville, KY 40202, USA; ^5^Markey Cancer Center, University of Kentucky, Lexington, KY 40536, USA; ^6^Center for Environmental & Systems Biochemistry, University of Kentucky, Lexington, KY 40536, USA; ^7^Institute for Biomedical Informatics, University of Kentucky, Lexington, KY 40536, USA

##### **Correspondence:** Hunter NB Moseley (hunter.moseley@uky.edu)

**Background**

**Fig. 1 (abstract P6).**
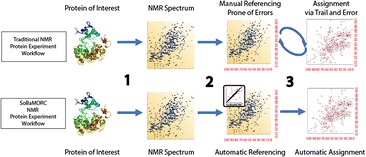
Traditional Protein NMR Referencing workflow leads to “chicken-egg dilemma” vs. proposed automatic Bayesian Model Optimized Reference Correction approach

Protein Nuclear Magnetic Resonance (NMR) plays an important role in the biophysical analysis of proteins, especially in the determination and study of their 3D structure. The accuracy of chemical-shifts assignments is a vital requirement for many aspects of NMR, especially protein structure determination. Traditional protein NMR technology relies on manual chemical shift referencing procedures that are prone to human error [Fig. 1] and cannot be validated until after the resonance assignment step. We present a Bayesian Model Optimized Reference Correction approach (BaMORC) that can provide correction to referencing before resonance assignment.

**Materials and methods**

We are developing a statistical-based algorithm to correct referencing by:

1. Computing composition probabilities of 20 amino acids of investigating protein C_α_ and C_β_ resonance pairs from the NMR data;
2. Summing the probabilities across all resonance pairs to give an estimate of amino acid (AA) composition; and
3. Minimize L1 errors between predicted and actual protein AA composition via a grid search method to estimate a minimum difference (correct referencing value) between.

**Results and conclusions**

**Fig. 2 (abstract P6).**
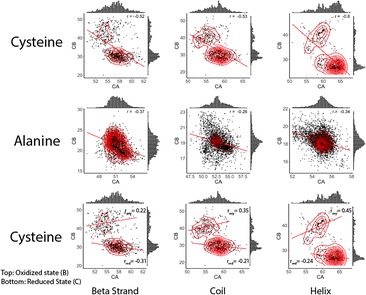
Example alpha and beta carbon shift bivariate distributions. C_α_ shifts are shown along the x-axis, while C_β_ shifts are shown along the y-axis. Distributions are shown cysteine distribution is dramatically different from the rest amino acid types, and treated it as single residue is incorrect. The bottom row shows the separated cysteine basing on oxidation states, which provide more usage to estimating reference of protein NMR

From our results, we identified that cysteine residues should be treated separately basing on its oxidized/reduced states [Fig. 2]. And the covariance between C_α_ and C_β_ resonance is a potent but long ignored statistic that should be utilized in the NMR referencing methodology. We have demonstrated that the overall approach is feasible. With applying BaMORC to the Re-referenced Protein Chemical shift Database RefDB [1], the 90% confidence range is 0.60 ppm, which suggest the estimated reference value is between -0.24 ppm and 0.45 ppm and assuming correct reference value is at 0 ppm.

Currently we are developing a shiny web app that will further simplify this protein NMR reference correction procedure. In the web interface, users can upload or paste their NMR peal list data directly into the app. The web app automatically groups the peaks into spin systems and applies the reference correction algorithm I have developed. The results of the analysis are returned as an html report and corrected peak list file.

The shiny web app will provide the biomolecular NMR field with a unique tool that allows NMR protein spectra referencing to be corrected and refined at the **beginning** of NMR protein experiments **without** using chemical shift assignments or protein 3D structure, which is the current retrospective referencing correction paradigm. Therefore, our method should improve both the speed and quality of protein resonance assignment and downstream NMR-based analyses including structure determination.

**Reference**

1. Haiyan Zhang, Stephen Neal and David Wishart (2003) "RefDB: A database of uniformly referenced protein chemical shifts" Journal of Biomolecular NMR, 25: 173-195.

## P7 MutChart: software application to assist with assessing veracity of candidate mutations

### Jerzy W Jaromczyk^1^, Mark Farman^2^, Li Chen^2^, Neil Moore^1^, Bailey K Phan^1^, Nathaniel J Serpico^1^, Ethan G Toney^1^

#### ^1^Department of Computer Science, University of Kentucky, Lexington, KY, 40508, USA; ^2^Department of Plant Pathology, University of Kentucky, Lexington, KY, 40546, USA

##### **Correspondence:** Jerzy W Jaromczyk (jurek@cs.uky.edu); Mark Farman (mark.farman@uky.edu)

**Background**

We present a bioinformatics application, MutChart, which streamlines the mutation identification and verification processes. We are using polymerase chain reaction-based random mutagenesis to generate a comprehensive library of mutations in the KCNH2 potassium channel gene that is responsible for ensuring proper heart rhythmicity. As part of this project, it is necessary to sequence a large number of PCR products to assess mutation density and spectrum. While candidate mutations can be identified by comparing the sequence data to a reference, each mutation should be manually validated to ensure its veracity.

**Materials and methods**

Many software programs are available for viewing raw sequence data for manual verification but none are designed in a way that facilitates high throughput visualization and validation steps. MutChart takes as input raw sequence trace data and the results of a blast search against the reference sequence. It then displays each mutation in a window that provides relevant information about the reference and alternate allele, the sequence quality score and, most importantly, a sequence trace plot for a few nucleotides on either side of the query nucleotide. The user views the trace plot to assess the candidate mutation’s veracity and then accepts the mutation, rejects it, or marks it as questionable. This action automatically advances the plot window to the next mutation, thereby eliminating the need for further user navigation.

**Conclusion**

MutChart is a result of a collaboration between computer scientists, who solved a number of challenges related to the processing and visualization of large datasets, and biologists who provided domain expertise in DNA sequence analysis and interpretation. Among interesting software solutions, the implementation utilizes caching to prevent from redundantly parsing previously used datasets, and uses dynamic loading to render the voluminous datasets.

**Acknowledgements**

J.W.Jaromczyk acknowledges support from the Bucks for Brains Program, Undergraduate Research Office at the University of Kentucky that funded the undergraduate students participating in this project.

## P8 Identifying heteroplasmy in *D. carota* using whole genome shotgun sequencing without known variants

### Caroline E Melton^1^, Jennifer R Mandel^1^, Bernie J Daigle, Jr.^1^

#### ^1^Department of Biological Sciences, The University of Memphis, Memphis, TN 38152, USA

##### **Correspondence:** Bernie J Daigle Jr (bjdaigle@memphis.edu)

**Background**

Organellar genomes are commonly inherited uniparentally, leading to a single genome being passed down without variation. Any recombination, biparental inheritance, or mutation of the organellar genomes leads to variation within the individual, known as heteroplasmy. Heteroplasmy has been observed in many species and is known to have phenotypic consequences, often resulting in reductions of fitness. In humans, it is associated with mitochondrial diseases and cancer. Research on the effects of heteroplasmy on the fitness of plants is limited, but studies suggest such genomic variation to be pervasive; wild carrot (*Daucus carota*) was found to be 60% heteroplasmic [1].

**Materials and methods**

MToolBox is an automated pipeline for the identification of heteroplasmy in humans that requires a complete human reference genome [2]. We have adapted this pipeline for more generalized use by allowing the input of any reference nuclear genome. Using a high-quality mapper (Bowtie 2), a duplicate marker (Picard Tools), and the assembler and VCF output generator from MToolBox, we are able to identify heteroplasmy frequencies and locations in a sample without requiring a reference of known heteroplasmic variants.

**Results**

Using whole genome shotgun (WGS) sequencing of four individuals of wild carrot, *D. carota*, we have identified high-confidence heteroplasmic sites in the mitochondrial and chloroplast genomes. Ongoing work involves searching for patterns of heteroplasmy within the population (*e.g.*, if it is more prevalent in exons or introns) and documenting the effects of heteroplasmy on fitness. In the future, we plan to scale up our analysis to over 190 samples of *D. carota*.

**References**

1. Mandel J, McCauley D**: Pervasive Mitochondrial Sequence Heteroplasmy in Natural Populations of Wild Carrot, Daucus carota spp. carota L.** *PLOS ONE* 2015, 10:e0136303.

2. Calabrese C, Simone D, Diroma M, Santorsola M, Gutta C, Gasparre G, Picardi E, Pesole G, Attimonelli M: **MToolBox: a highly automated pipeline for heteroplasmy annotation and prioritization analysis of human mitochondrial variants in high-throughput sequencing.** *Bioinformatics* 2014, 30:3115-3117.

## P9 Applying deep learning to predict phenotype based on genetic variation

### Hao Chen^1^ (hchen@uthsc.edu)

#### ^1^Department of Pharmacology, University of Tennessee Health Science Center, Memphis, TN 38103, USA

**Background**

Predicting phenotype based on genetic variation has long been a goal of genetic studies. Deep learning, including deep neural networks (DNN), has emerged as a superior method in many fields where machine learning was applied, such as image or speech recognition. Inspired by its success, We explored the potential of DNN in learning phenotype and genotype associations.

**Materials and methods**

We used a well-characterized data set of heterogeneous stock rats [1] that contained 1407 individuals and many phenotypes. We choose to focus on coat color because it has the most complete data and has a strong QTL, which is located on chr 1. We used the Keras library (ver 2.0.2) with the Theano backend (v 0.9.0) to train DNNs on the 46,943 chr 1 SNPs. A GPU (GeForce GTX 1070, 8 GB) running CUDA (8.0.61) was used to accelerate calculation.

**Results**

A simple neural network with one hidden layer of 200 neurons achieved an accuracy of 99.24% in predicting coat colors after 100 training epochs. Accuracy was reduced to 60.23% when new samples were tested, indicating model overfitting. Using five hidden layers increased test accuracy slightly to 61.93%. Further increasing the depth of the network reduced test accuracy. Adding dropout layers did not improve test accuracy. However, augmenting samples by swapping 20% of SNPs and then adding these swapped samples to the training set increased test accuracy to 63.07%. Test accuracy remained at 60.8% when the augmented samples were trained on a network with five hidden layers. In contrast to chr 1, the test accuracy was approximately 20% when these networks were trained on chr 2 data, which had no QTL for the phenotype. In summary, our data showed that DNN could learn genotype-phenotype associations directly from the raw genotype data. Further performance improvement likely will require much larger training data set. The code for this exercise is available in a GitHub repository (https://github.com/chen42/DNN4G2P/)

**References**

1. Rat Genome Sequencing and Mapping Consortium, et al. Combined sequence-based and genetic mapping analysis of complex traits in outbred rats. *Nat Genet* 2013, **45**(7): 767-775.

## P10 Evaluation of gene networks using literature cohesion

### Kazi I Zaman^1^, Ramin Homayouni^2,3^

#### ^1^Department of Computer Science, University of Memphis, Memphis, TN, 38152, USA; ^2^Department of Biological Sciences, University of Memphis, Memphis, TN, 38152, USA; ^3^Bioinformatics Program, University of Memphis, Memphis, TN, 38152, USA

##### **Correspondence:** Ramin Homayouni (rhomayon@memphis.edu)

**Background**

GeneNetwork [www.genenetwork.org] is a web tool that enables analysis of genetic and gene expression datasets across large panels of recombinant inbred mice [1]. Analysis of GeneNetwork data is challenging due to variability in microarray platforms, normalization methods, and biological factors. The goal of this project was to develop an analysis pipeline using literature-derived functional cohesion to evaluate GeneNetwork output and to extract meaningful insights.

**Materials and methods**

**Fig. 3 (abstract P10).**
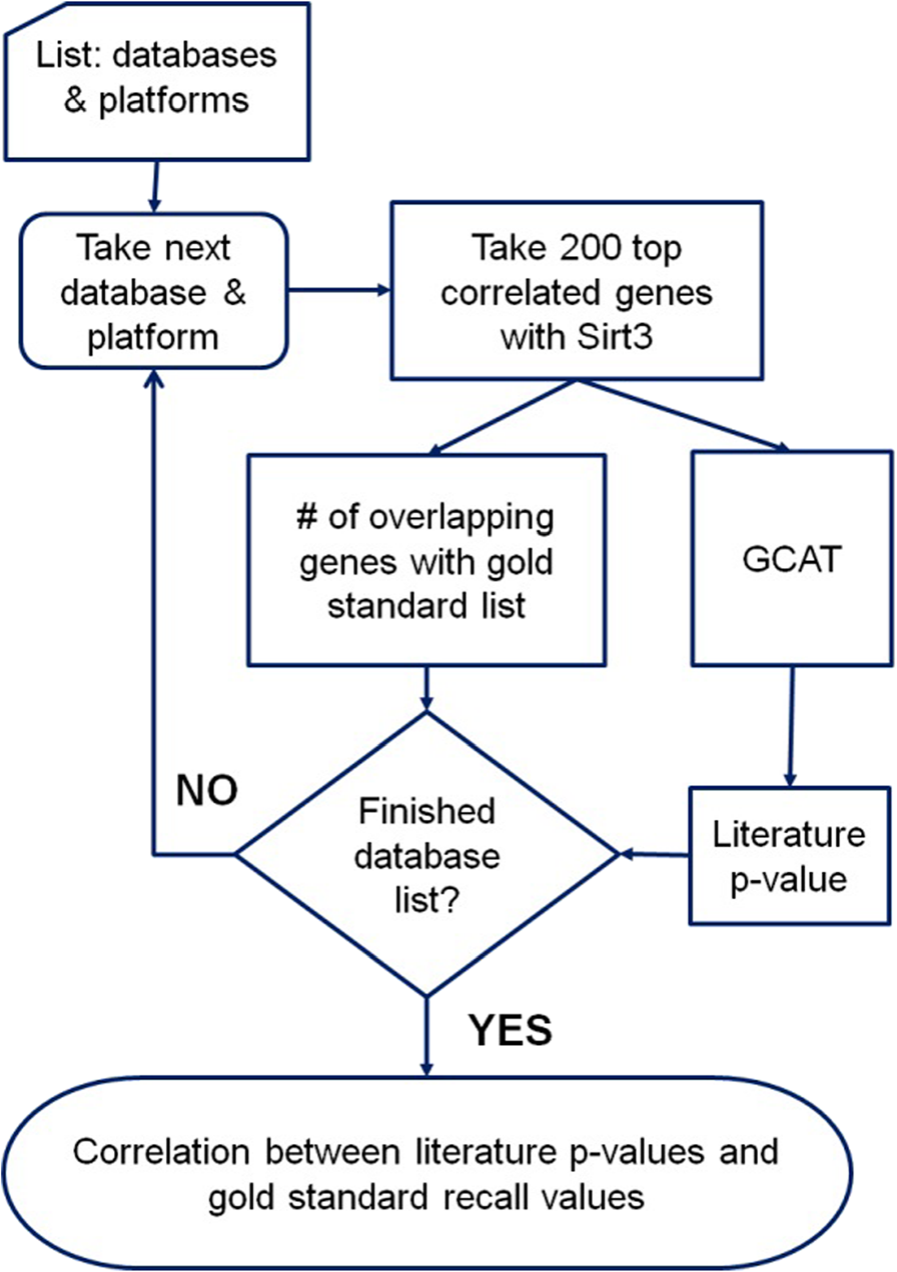
Analysis workflow

The workflow for our analysis pipeline is shown in Fig. 3. Using GeneNetwork, we identified the top 200 genes whose expression levels correlated with Sirt3 expression in liver tissues across BXD recombinant inbred mice. We examined Sirt3 correlated gene networks in seven liver datasets derived from different microarray platforms and normalization methods. For two datasets, two different Sirt3 probesets were analyzed. Literature cohesion p-values (LPv) were calculated for the top 200 Sirt3 correlated genes using GeneSet Cohesion Analysis Tool [http://binf1.memphis.edu/gcat/] that was developed by our group previously [2]. To evaluate our approach, we used a gold-standard set of 429 Sirt3 target proteins, which were previously reported to be differentially acetylated in liver tissues from Sirt3 knockout mice compared with wildtype controls [3]. Recall refers to the number of overlapping genes between Sirt3-correlated gene network and the gold-standard set. Functional enrichment analysis was performed using DAVID [https://david.ncifcrf.gov/].

**Results**

**Fig. 4 (abstract P10).**
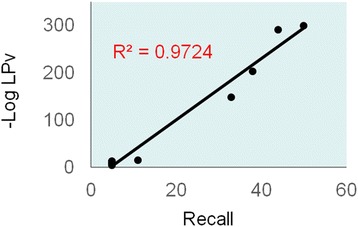
Correlation between literature cohesion and gold-standard gene recall for Sirt3 gene networks in liver. Literature cohesion p-value (LPv) and recall were calculated for nine Sirt3 gene networks (each included 200 Sirt3 correlated genes) derived from seven different BXD recombinant inbred mouse datasets

We found a very high correlation (R^2^ = 0.97) between literature cohesion of Sirt3-correlated gene networks and recall of the gold-standard set (Fig. 4). Functional enrichment analysis of the network with the lowest LPv revealed that the Sirt3 correlated genes belong to the following Gene Ontology classifications among many others: Mitochondrion (p-value = 4.3E-42), Oxidoreductase Activity (p-value = 2.3E-40), Lipid Metabolism (p-value = 1.2E-12), and Synthesis of Amino Acid (p-value = 1.7E-7). These results are consistent with previous reports that Sirt3 is a key regulator of mitochondrial metabolic processes [3].

**Conclusions**

Our results provide proof-of-concept that literature cohesion analysis can rapidly identify biologically meaningful gene networks from the vast amount of genomic data accumulating in publicly available resources such as Genenetwork.org and Gene Expression Omnibus (GEO). We posit that our approach will facilitate discovery from high throughput genomic data.

**References**

1. Chesler EJ, Lu L, Shou S, Qu Y, Gu J, Wang J, Hsu HC, Mountz JD, Baldwin NE, Langston MA, Threadgill DW, Manly KF, Williams RW: **Complex trait analysis of gene expression uncovers polygenic and pleotropic networks that modulate nervous system function.** *Nat Genet* 2005, **37**:233-242.

2. Xu L, Furlotte N, Lin Y, Heinrich K, Berry MW, George EO, Homayouni R: **Functional cohesion of gene sets determined by latent semantic indexing of PubMed abstracts.** *PLoS One* 2011, **6**: e18851.

3. Rardin MJ, Newman JC, Held JM, Cusack MP, Sorensen DJ, Li B, Schilling B, Mooney SD, Kahn CR, Verdin E, Gibson BW: **Label-free quantitative proteomics of the lysine acetylome in mitochondria identifies substrates of SIRT3 in metabolic pathways."** *Proc Natl Acad Sci USA* 2013, **110**: 6601-6606.

## P11 Molecular fingerprinting for inferring a Gaussian Graphical Model representation of a stable coronary artery disease plasma interactome using adaptive graphical Lasso penalization

### Patrick J Trainor^1,2*^, Samantha M Carlisle^3^, Andrew P DeFilippis^1,2§^, Shesh N Rai^4,5§^

#### ^1^Department of Medicine, Division of Cardiovascular Medicine, University of Louisville, Louisville, KY 40202 USA; ^2^Diabetes and Obesity Center, University of Louisville, Louisville, KY 40202 USA; ^3^Department of Pharmacology and Toxicology, University of Louisville, Louisville, KY 40202 USA; ^4^Biostatistics Shared Facility, James Graham Brown Cancer Center, University of Louisville, Louisville, KY 40202 USA; ^5^Department of Bioinformatics and Biostatistics, University of Louisville, Louisville, KY 40202 USA

##### **Correspondence:** Patrick J Trainor (patrick.trainor@louisville.edu)

^§^Equally contributing authors

**Background**

As disease states are either precipitated by or result in metabolic dysregulation, metabolite concentrations can be utilized for determining physiological processes that are differentially impacted across disease states. For example, while coagulation is a homeostatic response to vascular injury, dysregulation can lead to pathological thrombosis, the cause of acute myocardial infarction—a leading cause of death in humans. To determine such dysregulation a representation of the metabolome in a non-pathological state or a reference phenotype is needed. We sought a Gaussian Graphical Modeling (GGM) approach for constructing a reference metabolome that incorporates prior knowledge of biochemical structural similarity. A full joint distribution representation was sought to facilitate inference regarding partial correlation structure. We evaluated the method for constructing a plasma metabolome for a stable, yet diseased state from human subjects presenting with Coronary Artery Disease (CAD). This representation will provide a reference for systems-level comparisons across the disease state transition from stable to acute myocardial infarction.

**Materials and methods**

The Graphical Lasso (gLASSO: graphical least absolute shrinkage and selection operator) was proposed for the estimation of sparse inverse covariance matrices for multivariate Gaussian distributions. The gLASSO algorithm estimates the inverse of the covariance matrix by maximizing the L1 penalized log-likelihood function via coordinate descent. Ambroise, et al. proposed modifying the regularization term to incorporate an adaptive penalty. In previous applications, the adaptive penalization for estimating concentration matrices was predicated on assuming a latent clustering of the variables, to be estimated by expectation maximization or other clustering approaches. We instead devise an adaptive penalty that varies inversely with molecular similarity. Molecular similarity was defined via the Tanimoto distance measure using bitwise atom-pair fingerprinting and was used for generating adaptive penalties in constructing a plasma metabolome for the phenotype of interest.

**Results**

We constructed a reference plasma metabolome for a CAD phenotype. We observed that for a fixed number of edges in the Gaussian Graphical Model (GGM). As expected, adaptive penalization increased the likelihood of edge was formation between metabolites that are structurally related.

**Conclusions**

While our evaluation does not provide evidence that biochemical fingerprint-based adaptive penalization increases the overall likelihood of a GGM in representing a metabolome, a theoretical evaluation is needed. A framework for the probabilistic integration of prior biochemical knowledge in constructing metabolomics-based graphical models remains desirable for facilitating pathway and biochemical process level inference.

