# Correction to: Proceedings of the 16th Annual UT-KBRIN Bioinformatics Summit 2017: bioinformatics

**DOI:** 10.1186/s12859-017-1880-9

**Published:** 2017-11-15

**Authors:** Eric C. Rouchka, Julia L. Chariker, David Tieri, Juw W. Park

**Affiliations:** 1 Department of Computer Engineering and Computer Science, Speed School of Engineering, University of Louisville, Louisville, KY 40292 USA; 2 Department of Psychological and Brain Sciences, College of Arts and Sciences, University of Louisville, Louisville, KY 40292 USA; 3 Department of Anatomical Sciences and Neurobiology, University of Louisville, Louisville, KY 40292 USA

## Correction

After publication of this supplement [1], it was brought to our attention that the wrong year was used in the title of the supplement. The 16th Annual UT-KBRIN Bioinformatics Summit was held in 2017.
